# Retinoic acid signaling in heart development: Application in the differentiation of cardiovascular lineages from human pluripotent stem cells

**DOI:** 10.1016/j.stemcr.2021.09.010

**Published:** 2021-10-14

**Authors:** Alexandra Wiesinger, Gerard J.J. Boink, Vincent M. Christoffels, Harsha D. Devalla

**Affiliations:** 1 Department of Medical Biology, Amsterdam University Medical Centers, Meibergdreef 9, 1105 AZ Amsterdam, the Netherlands; 2 Department of Cardiology, Amsterdam University Medical Centers, Meibergdreef 9, 1105 AZ Amsterdam, the Netherlands

## Abstract

Retinoic acid (RA) signaling plays an important role during heart development in establishing anteroposterior polarity, formation of inflow and outflow tract progenitors, and growth of the ventricular compact wall. RA is also utilized as a key ingredient in protocols designed for generating cardiac cell types from pluripotent stem cells (PSCs). This review discusses the role of RA in cardiogenesis, currently available protocols that employ RA for differentiation of various cardiovascular lineages, and plausible transcriptional mechanisms underlying this fate specification. These insights will inform further development of desired cardiac cell types from human PSCs and their application in preclinical and clinical research.

## Teaser

In this article, Devalla and colleagues outline the role of retinoic acid (RA) signaling in the heart and provide an overview of human pluripotent stem cell-based cardiac differentiation approaches that employ RA. They offer new insights into molecular mechanisms underpinning the function of RA signaling and discuss the crosstalk with wingless-related integration site (WNT) and bone morphogenetic protein (BMP) signaling in cardiac cell fate specification.

## Introduction

**Figure 1:**
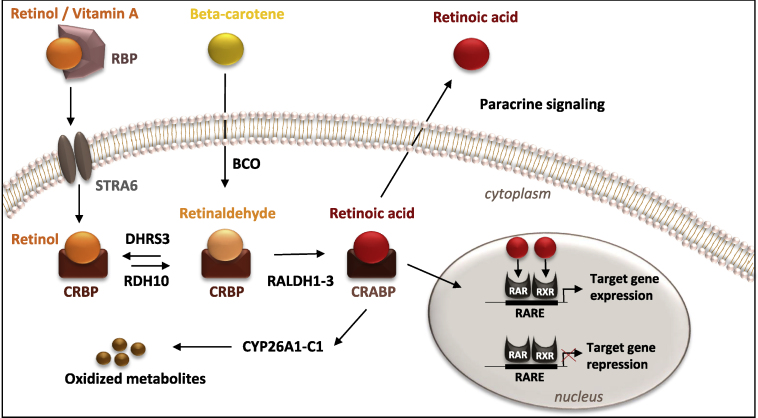
Biosynthesis and metabolism of RA in vertebrates Retinol obtained from diet is converted to retinaldehyde by retinal dehydrogenase enzymes. Retinaldehyde can also be synthesized from β-carotene. Oxidation of retinaldehyde to RA is carried out by RALDH enzymes. RA is a diffusible morphogen with autocrine as well as paracrine actions. RA is the ligand for heteromeric RAR and RXR receptors, which bind to RARE in promoters of target genes. In non-target tissues, RA is degraded by CYP26 enzymes.

Retinoic acid (RA), the active derivative of vitamin A, has pleiotropic functions during vertebrate development, differentiation, and homeostasis. Vitamin A is obtained from diet, either as preformed vitamin A (retinol, retinyl esters) found in animal products or precursors such as carotenoids found in plant products. Retinol is bound by plasma retinol binding protein and transthyretin complexes for transport to target tissues, where it is taken up through STRA6 cell-surface receptors (Kanai et al., 1968; Kawaguchi et al., 2007). Once inside the cell, retinol forms a complex with intracellular retinol binding proteins (Napoli, 2016), and is processed by short-chain dehydrogenase/reductase, RDH10, for conversion to retinaldehyde (Sandell et al., 2007). Retinaldehyde can be reconverted back to retinol by DHRS3 for storage, which helps in maintaining RA homeostasis (Billings et al., 2013). Oxidation of retinaldehyde to RA is carried out by retinaldehyde dehydrogenases (RALDH1–3, also known as ALDH1A1–3) (Duester, 2008). Among RALDH enzymes, RALDH2 is the major source of RA during embryogenesis, and its expression was shown to closely correlate with active RA signaling (Niederreither et al., 1997; Moss et al., 1998). RA uptake is mediated by binding to cellular RA binding proteins, CRABP, which deliver RA to the nucleus for regulation of gene expression or to CYP26 enzymes (CYP26A1, CYP26B1, and CYP26C1) for degradation. Biosynthesis and metabolism of RA is briefly summarized in Figure 1 and extensively reviewed elsewhere (Kedishvili, 2016; Metzler and Sandell, 2016).

Vitamin A is an essential nutrient required throughout life. Notably, RA, the physiologically active form of vitamin A, is critical for proper development of the embryo. The majority of our knowledge regarding the consequences of maternal vitamin A deficiency and the impact on fetal development came from studies in animal models. A wide spectrum of multi-organ abnormalities including those of the heart were observed in the progeny of vitamin A-deficient rats (Wilson et al., 1953; Wilson and Warkany, 1949). In humans, congenital heart defects have been reported in people carrying mutations in *STRA6* and *RALDH2*, which results in RA deficiency (Pasutto et al., 2007; Pavan et al., 2009). Importantly, an excess of vitamin A during pregnancy has also been associated with heart defects among other malformations and was even found to be teratogenic (Lammer et al., 1985; Rothman et al., 1995). These findings suggest that a precise balance in RA availability underscores normal development.

The functions of RA in embryogenesis mainly stem from its role in gene regulation. RA acts as a ligand for members of the steroid hormone superfamily of transcription factors, the retinoic acid receptors (RARs) and the retinoic acid X receptors (RXRs). Both receptor subfamilies comprise three independent genes: RARα, RARβ, RARγ and RXRα, RXRβ, RXRγ. While RA is the sole ligand for RARs, RXRs can also be bound by other ligands such as polyunsaturated fatty acids (Dawson and Xia, 2012). RARs and RXRs form heterodimers and bind to RA-response element (RARE) in the regulatory regions (promoters, enhancers) of target genes and recruit co-activators or co-repressors to regulate gene expression in a ligand-dependent manner. In this review, we focus on the role of RA in heart development, particularly in the specification of various cardiac cell types and its application in differentiating these cell populations from human pluripotent stem cells (hPSCs).

## Overview of RA signaling in cardiogenesis

Heart development begins at gastrulation, when cell populations in the primitive streak are patterned to form distinct cardiac cell types (Devine et al., 2014; Lescroart et al., 2014; Ivanovitch et al., 2021). Left ventricular progenitors are the first to emerge from distal primitive streak at mid-streak stage (between embryonic day 7 [E7.0] and E7.75), followed by right ventricular progenitors at late-streak stage (E7.25–E7.75) and outflow tract (OFT) progenitors at no-allantoic-bud to early-allantoic-bud stages (E7.25–E8.0). Atrial progenitors leave the primitive streak concomitant with OFT progenitors, albeit from a more proximal region (Ivanovitch et al., 2021). The cardiac progenitors migrate from the primitive streak to the anterior lateral plate mesoderm and form bilateral fields, termed the first heart field (FHF) and the second heart field (SHF). The FHF cells express *Hand1* and *Hcn4* among other markers. Recently, an additional multipotent progenitor field anterior to the FHF, also expressing *Hand1*, has been identified to contribute to myocardium of the left ventricle, atrioventricular canal, and the proepicardium (Tyser et al., 2021; Zhang et al., 2021). While the FHF differentiates and forms the linear heart tube, the SHF is maintained in a proliferative progenitor state (Prall et al., 2007). Subsequently, cells of the SHF are progressively added to the heart tube. The SHF dorsal of the forming heart tube is partitioned into an anterior component (aSHF) marked by *Tbx1* and a posterior component (pSHF) marked by *Tbx5* (Bruneau et al., 1999; De Bono et al., 2018). The aSHF progenitors of the right ventricle and outflow tract are contiguous with the arterial pole of the heart tube, and pSHF progenitors of the atria and sinus venosus are connected to the venous pole of the linear heart tube. The elongating heart undergoes rightward looping. Simultaneously, the proepicardium develops caudoventrally of the inflow tract, undergoes epithelial-to-mesenchymal transition (EMT), and forms the epicardium, the outer layer of the heart.

**Figure 2:**
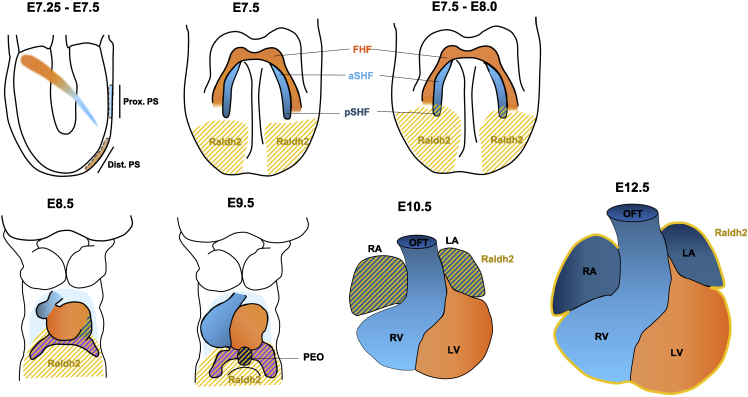
Expression of *Raldh2* in the developing mouse heart Cardiac progenitors migrate anterolaterally from the primitive streak. *Raldh2 is* expressed in the posterior part of the embryo at E7.5, which expands anteriorly, toward the cardiogenic fields. Thereafter, the pSHF progenitors express *Raldh2* and the expression is found in the sinus venosus, atria, and the proepicardium at E9.5. Raldh2 expression is restricted to the atrial myocardium at E10.5–E11.5 and to the epicardium at E12.5.

RA signaling has been implicated in multiple stages of heart development including formation of anterior-posterior boundaries of cardiac mesoderm, specification of cardiomyocyte subtypes, development of the epicardium, the outflow tract, and growth of the ventricular compact wall and coronary arteriogenesis. Early studies investigating the expression of *Raldh2* (Figure 2), and the effects of its deletion provided insights into spatiotemporal requirements of RA in the developing heart. At primitive-streak to early-bud stages, cardiac progenitors do not express *Raldh2* (Ivanovitch et al., 2021). At late-bud stages (E7.5–E8.0), *Raldh2* is expressed in the posterior lateral plate mesoderm and expands anteriorly, encountering caudal cardiac precursors at early head fold stage (E7.5–E8.0) (Niederreither et al., 1997; Hochgreb et al., 2003). Subsequently, *Raldh2* is expressed by pSHF progenitors themselves and remains confined to the prospective sinoatrial and atrial tissues until E9.5 (Moss et al., 1998; Hochgreb et al., 2003; Ivanovitch et al., 2021). At E9.5, the proepicardium is formed, which also shows robust expression of *Raldh2* (Del Monte et al., 2011). At E10.5 and E11.5, *Raldh2* expression persists in the atrial myocardium. At E12.5, *Raldh2* expression is restricted to the epicardium of both the atria and the ventricles (Moss et al., 1998; Brade et al., 2011), and gradually decreases at late gestational stages.

The balance between RA synthesis by RALDH2 and degradation by CYP26 enzymes determines RA availability and distribution in the embryo. Interestingly, *Raldh2* and *Cyp26* expression domains are largely complementary (Niederreither and Dollé, 2008). In the heart, *Cyp26a1* is expressed in the endocardium of the outflow tract, atria, and sinus venosus between E8.0 and E9.0 (MacLean et al., 2001). At E14.5, *Cyp26a1* expression is restricted to atrioventricular valves, whereas *Cyp26b1* is expressed in the endocardium of the outflow tract (Abu-Abed et al., 2002). Unlike the strict expression patterns of RALDH2 and CYP26 enzymes, RA receptors are expressed widely. In particular, *Rarα* and all three *Rxr* genes show ubiquitous expression in the developing heart. *Rarb1* expression is restricted to the outflow tract mesenchyme, while *Rarb2* is found throughout the developing myocardium. *Rarγ* is specifically expressed in the endocardial cushions and developing vessels at E12.5 (reviewed in Dollé, 2009). Insights on RA signaling obtained from experimental animal models greatly contributed to the understanding of organ development and have been successfully applied to *in vitro* differentiation protocols discussed below (Zhang et al., 2011; Devalla et al., 2015; Iyer et al., 2015; Guadix et al., 2017; Lee et al., 2017; Protze et al., 2017; Zhao et al., 2017).

## RA in atrial development

The majority of the atrial compartments are formed by contributions from the pSHF progenitors. While the caudal pSHF gives rise to the sinus venosus and distal atria, cranial sections of the pSHF form the proximal portions of the atria closer to the atrioventricular junction (Domínguez et al., 2012). Left-right identity is conferred by *Pitx2c* expression in the left pSHF, which contributes to most of the left atrium (Galli et al., 2008). RA signaling is instrumental in maintaining the posterior limits of the SHF (Ryckebusch et al., 2008). Moreover, the boundary between aSHF and pSHF is facilitated by the expression of RA-responsive Hox genes as well as T-box transcription factors *Tbx1* and *Tbx5* (Bertrand et al., 2011; De Bono et al., 2018; Stefanovic et al., 2020). While Tbx1 expressed in aSHF antagonizes RA signaling, RA-induced Tbx5 in the pSHF is required to suppress the Tbx1-dependent aSHF program (De Bono et al., 2018). Abrogation of RA signaling in *Raldh2* knockout mice results in hypoplastic atria and sinus venosus (Niederreither et al., 2001), while a more dramatic total lack of the sinus venosus was observed in vitamin A-deficient quail embryos (Kostetskii et al., 1999). Similarly, inhibition of RA signaling in Hamburger-Hamilton stage 4–7 chicken embryos using a pan RA receptor antagonist resulted in a smaller inflow tract compartment and enlarged ventricles (Hochgreb et al., 2003).

**Figure 3:**
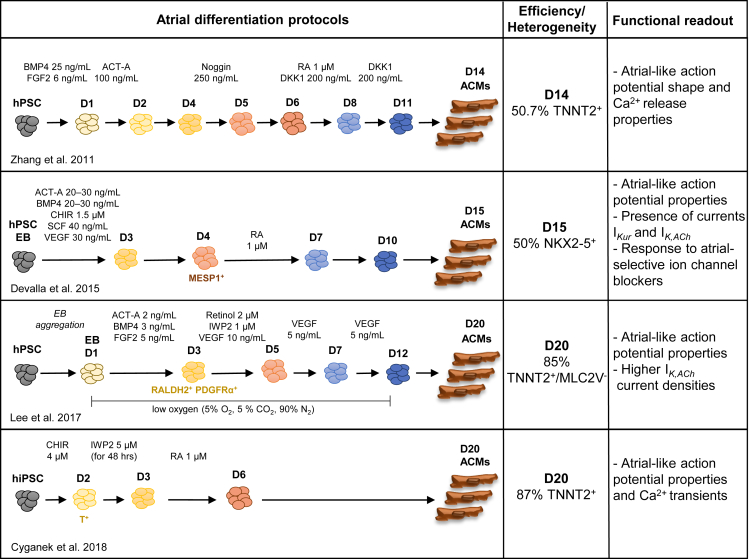
Summary of differentiation protocols for the generation of atrial cardiomyocytes from hPSCs

Consistent with *in vivo* studies, the role of RA in atrial differentiation has also been investigated and confirmed in several *in vitro* studies (Gassanov et al., 2008; Zhang et al., 2011; Devalla et al., 2015; Lee et al., 2017). The first report was based on experiments with transgenic mouse embryonic stem cells (mESCs), in which GFP expression was driven by the atrial-specific promoter of *Nppa* (Wobus et al., 1997; Gassanov et al., 2008). The authors examined the effect of varying concentrations of RA treatment for 5 days, during early or late stages of differentiation. They concluded that embryoid bodies treated with 100 nM and 1 nM RA from day 1–6 differentiated toward an atrial phenotype as assessed by an increase in Nppa-GFP^+^ areas. However, this increase was a moderate 6%–9% in the RA-treated group, and the changes in *Myl7* (*Mlc2a*) and *Myl2* (*Mlc2v*) mRNA expression were only significant in the 100 nM condition. Nonetheless, this study indicated a possible role for RA in atrial differentiation *in vitro*. Subsequently, a role for RA in directing atrial specification of human embryonic stem cells (hESCs) was described (Zhang et al., 2011). In this study, upon mesoderm formation, bone morphogenetic protein (BMP) signaling was inhibited using Noggin, followed by treatment with 1 μM RA and wingless-related integration site (WNT) inhibitor DKK1. The differentiation timeline suggested that RA treatment was performed at the cardiac progenitor stage in this approach; however, corroborating data are not available. In the study by Zhang et al., atrial versus ventricular phenotype of resulting cells was primarily assessed by a decrease in MLC2V and an increase in MLC2A, as well as ANP protein expression. Furthermore, action potential shape and calcium transient properties were used to differentiate atrial and ventricular cardiomyocytes. Studies performed in hPSCs are summarized in Figure 3.

Further evidence for RA-driven atrial specification in hESCs was provided by our group (Devalla et al., 2015). In contrast to the study by Zhang et al. (2011), we found that atrial differentiation relies on the stimulation of RA signaling shortly after the peak expression of *MESP1* at the cardiac mesoderm stage. In agreement with concentration-dependent effects of RA, we observed that treatment with a lower concentration of RA (1–10 nM) improved differentiation efficiency but had no effect on subtype specification. This is consistent with findings in mESCs that 1–10 nM RA accelerates cardiac differentiation (Wobus et al., 1997). However, treatment with a higher concentration of RA (1 μM) at late mesoderm stage (day 4) steered differentiation toward atrial fate. Moreover, we identified that atrial-specific genes *NR2F1* (*COUP-TFI*) and *NR2F2* (*COUP-TFII*) are induced in response to RA, as also observed in zebrafish mesoderm (Dohn et al., 2019). These transcription factors bind directly to regulatory DNA sequences of ion channel genes *KCNA5* and *KCNJ3*, which confer unique electrophysiological properties to atrial cells. In addition, *in vivo* studies identified a role for *Nr2f2* in maintaining atrial chamber identity and size (Wu et al., 2013; Duong et al., 2018).

RA-directed atrial fate specification *in vitro* has been shown to be orchestrated by MEIS2, which antagonizes ISL1, in order to induce the atrial transcription factor, *NR2F1* (Quaranta et al., 2018). Mounting evidence suggests that the TALE family of genes such as MEIS1/2 and PBX1–3 are co-factors for HOX-mediated gene regulation (reviewed in Lescroart and Zaffran, 2018). Indeed, putative enhancer regions identified in pSHF cells revealed enrichment for HOX and TALE binding motifs (Stefanovic et al., 2020). While HOX and MEIS genes appear to be direct targets of RA itself (Mercader et al., 2000; Berenguer et al., 2020), studies have shown that they can also regulate *Raldh2* expression and thereby RA levels through a positive feedback loop (Vitobello et al., 2011; López-Delgado et al., 2021). Interestingly, misexpression of *Hoxb1* in the aSHF domain resulted in an upregulation of *Tbx5* and *Nr2f2* (Stefanovic et al., 2020), both of which are implicated in the differentiation of atrial and sinoatrial nodal cells *in vivo* and *in vitro* (Liberatore et al., 2000; Wu et al., 2013; Devalla et al., 2015; Protze et al., 2017). These findings suggest that co-operative binding of HOX and MEIS proteins to target genes is one of the earliest events directing specification toward atrial and sinoatrial lineages.

Current protocols yield 50%–90% TNNT2^+^ cells in atrial differentiations. Analyses of these cardiomyocytes by genetic marker expression, such as NR2F2 or by action potential morphology, indicate that a large majority of these cardiomyocytes have an atrial-like phenotype. Nonetheless, the expression of *SHOX2* in cells obtained from atrial differentiations suggests the presence of a sinoatrial node-like cardiomyocyte (SANCM) side population in these cultures (Devalla et al., 2015; Cyganek et al., 2018). To improve efficiencies across various cell lines, it is important to better characterize the differentiation process. Besides optimizing timing and concentration of RA application, the induction of appropriate mesoderm has been proposed to regulate differentiation of hPSCs to atrial versus ventricular identities. The *RALDH2-*expressing mesodermal population has been shown to have a higher potential for atrial fate compared with *CYP26A1* (enzyme that degrades RA)-expressing mesoderm, which is better suited for differentiation toward ventricular lineage (Lee et al., 2017). Even though exogenous RA induced >90% TNNT2^+^ cells from *CYP26A1*^+^ mesoderm, expression of atrial genes was lower than in *RALDH2*^+^ mesoderm treated with RA. Furthermore, *RALDH2*^+^ mesodermal cells treated with 2 μM retinol generated 85% TNNT2^+^ cells, suggesting that they efficiently produce RA required for differentiation toward the atrial lineage. Nevertheless, reduction in the expression of the ventricular gene *MYL2* and increase in expression of the atrial gene *KCNJ3* were more pronounced upon treatment with RA itself.

Due to applications in drug screening and disease modeling for atrial fibrillation, there has been a lot of interest in hPSC-derived atrial cells. To fully utilize the potential of these cells, further refinement of their phenotype is required. For example, hPSC-atrial cells exhibit relatively faster beating rates (about 1–1.2 Hz), comparable with hPSC-derived SANCMs, reflecting their immaturity (Devalla et al., 2015; Lee et al., 2017; Protze et al., 2017). Strategies to promote maturation of hPSC-atrial cells are thus important. Moreover, the right versus left atrial identity of these cells has not been evaluated. Right and left atrial cardiomyocytes display differences in gene expression as well as propensity for atrial fibrillation (Van Ouwerkerk et al., 2020). Directed approaches to generate right versus left atrial cells will open avenues for application of these cells in better understanding the etiology of atrial fibrillation.

## RA in sinoatrial node development

The sinoatrial node (SAN) is referred to as the pacemaker of the heart, as it generates electrical impulses that set the rate and rhythm of cardiac contractions. At E7.5 in mice and 16- to 23-somite stage in zebrafish, a distinct progenitor population at the lateralmost edge of the pSHF downregulates *Nkx2-5* and initiates *Tbx18* expression (Mommersteeg et al., 2010; Ren et al., 2019). These progenitors also express *Isl1* and are subsequently integrated into the differentiating sinus venosus myocardium at the inflow tract of the expanding heart tube (Christoffels et al., 2006; Mommersteeg et al., 2010). Following looping and remodeling of the linear heart tube, the SAN is established at the entrance of the superior caval vein in the right atrium and takes over as the primary pacemaker at E12.5 in mouse (Yi et al., 2012). The SAN is a structurally and functionally complex tissue, which can be broadly divided into three regions: the head region marked by both *Tbx18* and *Tbx3* and conspicuous by the absence of *Nkx2–5*; the tail region marked by the presence of *Tbx3* and *Nkx2–5* but devoid of *Tbx18* (Wiese et al., 2009; Goodyer et al., 2019; Li et al., 2019); and a transition zone with a molecular and functional phenotype intermediate to that of SAN and the adjacent atrial tissue (Boyett et al., 2000; Goodyer et al., 2019; Li et al., 2019).

**Figure 4:**
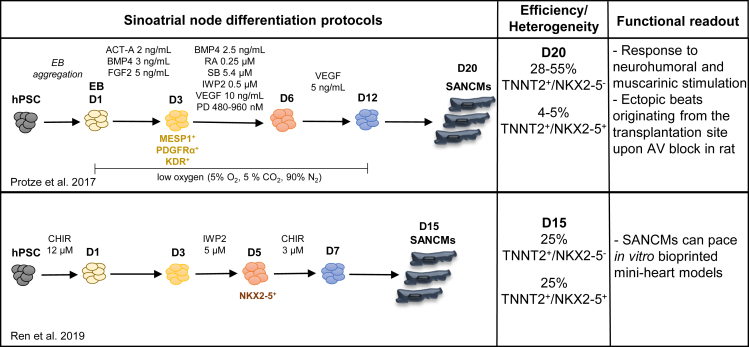
Summary of differentiation protocols for the generation of sinoatrial nodal cardiomyocytes from hPSCs

As discussed above, RA signaling is important for the development of atria and sinus venosus. In line with this, RA has also been implicated in the *in vitro* differentiation of hPSCs to SANCMs (Protze et al., 2017). Treatment of *MESP1*^+^ cardiac mesoderm with BMP4 and RA (hereafter BMP4 + RA) resulted in cardiomyocytes with SAN-like molecular and functional profile (Figure 4). The early requirement for BMP and RA signaling in the differentiation of inflow tract myocardium is evidenced by several studies. BMP ligands are involved in the recruitment of pSHF toward the myocardial lineage (Kruithof et al., 2006; Schlueter et al., 2006; van Wijk et al., 2009). Similarly, atrial and SAN progenitors in the pSHF are progressively exposed to RA. The *Raldh2-*expressing field is initially in close proximity to the pSHF progenitors followed by the expression of *Raldh2* in the cardiac progenitor cells themselves (Hochgreb et al., 2003). While it is conceivable that the pSHF progenitors first receive RA via a paracrine action, RARE-lacZ data at these early stages is not available. Nevertheless, pharmacological inhibition of RA signaling in chicken embryos, using an RAR antagonist, revealed an early requirement for RA signaling in the pSHF, even before the onset of *Raldh2* expression (Hochgreb et al., 2003).

Although application of BMP4 alone at the cardiac mesoderm stage was sufficient to generate the majority of NKX2–5^−^ SAN cardiomyocyte population, addition of RA was crucial to enhance the expression of SAN associated genes such as *TBX5* (Protze et al., 2017). This is in accordance with *in vivo* findings that have demonstrated an association between RA and *Tbx5* (Liberatore et al., 2000; De Bono et al., 2018). During embryonic development, *Raldh2* is co-expressed with *Tbx5* in a subset of pSHF progenitors (Hochgreb et al., 2003; Ryckebusch et al., 2008; De Bono et al., 2018), and Raldh2 knockouts display reduced *Tbx5* expression (Niederreither et al., 2001; Sirbu et al., 2008). Furthermore, a recent study revealed that *Tbx5* promotes pSHF identity through a positive feedback loop with *Raldh2* (Rankin et al., 2021). Importantly, Tbx5 regulates the expression of a key pacemaker gene, *Shox2* (Puskaric et al., 2010), which is in turn implicated in regulating other important SAN genes such as *Bmp4* and *Isl1* (Puskaric et al., 2010; Hoffmann et al., 2013). These findings suggest that RA promotes pacemaker identity by establishing an SAN gene-regulatory network via TBX5.

Interestingly, both in limb and lung development, where Tbx5 plays an important role, the transcription factor interacts with canonical WNT signaling. Whereas in limb development canonical WNT signaling is upstream of *Tbx5*, in lung development Tbx5 activates the expression of *Wnt2* in cardiopulmonary progenitors (Ng et al., 2002; Nishimoto et al., 2015; Steimle et al., 2018; Rankin et al., 2021). WNT signaling has also been identified as a critical cue for pacemaker development (Bressan et al., 2013; Ren et al., 2019). An alternative approach described for the differentiation of hPSCs to SAN cardiomyocytes showed that the activation of canonical WNT signaling is sufficient to enforce pacemaker identity (Ren et al., 2019) (Figure 4). One of the key differences between the BMP4 + RA and the WNT protocols is the timing of signaling modulation. While BMP4 + RA is applied at the cardiac mesoderm stage (*MESP1*^+^), activation of canonical WNT signaling is performed at the cardiac progenitor stage (*NKX2*–*5*^+^). It is also noteworthy that BMP4 + RA treatment is performed in conjunction with inhibition of WNT signaling using small-molecule inhibitors such as IWP2. During early stages of cardiogenesis, a biphasic antagonistic role for WNT signaling has been identified (Naito et al., 2006; Ueno et al., 2007). These processes are simulated in cardiac differentiation protocols, where WNT signaling is first activated in hPSCs and subsequently inhibited upon cardiac mesoderm induction to promote cardiomyocyte formation. Following the inhibition of WNT signaling at cardiac mesoderm stage, which results in *NKX2*–*5*^+^ cardiac progenitors, canonical WNT signaling was activated to direct cells toward the SAN lineage.

Similar to the BMP4 + RA protocol for SANCM differentiation (Protze et al., 2017), the WNT protocol also resulted in cardiomyocytes expressing key SAN genes such as *SHOX2*, *ISL1*, and *BMP4* (Ren et al., 2019). *Bmp4*, as well as *Isl1*, have been identified as downstream targets of canonical WNT signaling in cardiac progenitors (Klaus et al., 2007; Lin et al., 2007), which likely mediate the differentiation toward pacemaker cells along with Tbx5, as discussed above. Interestingly, Tbx5 and Bmp4 are also crucial for the specification of the proepicardium (Liu and Stainier, 2010). Previous studies have shown that the proepicardial cells and the myocardial cells of the inflow tract diversify from a common progenitor population (van Wijk et al., 2009; Mommersteeg et al., 2010). Comparably, the non-cardiomyocyte population obtained from both the BMP4 + RA and the WNT differentiation protocols is of epicardial identity (Ren et al., 2019; A.W., and H.D.D., unpublished observations). Further research is needed to evaluate the crosstalk between BMP, RA, and WNT signaling in SANCM and epicardial differentiation.

As the SAN is composed of several subpopulations, it remains to be evaluated whether both BMP4 + RA and WNT differentiation protocols generate the same cellular subtypes of the SAN. It has recently been demonstrated that a subpopulation of the SAN expressing *Shox2* and *Nkx2–5* (“SAN junction cells” of the transitional zone) plays a crucial role in atrial activation (Li et al., 2019). Both differentiation approaches generate around 55% of TNNT2^+^ cardiomyocytes (CMs). However, only about half of the TNNT2^+^ cells are NKX2–5^−^ in the WNT-based protocol (Ren et al., 2019), a gene expression pattern found in SAN head cells *in vivo* (Wiese et al., 2009; Goodyer et al., 2019). Whether the remaining NKX2–5^+^ cardiomyocyte fraction corresponds to SAN transitional zone populations is not known (Ren et al., 2019). On the other hand, the BMP4 + RA-based protocol results in 55% NKX2–5^−^ CMs and only 5% NKX2–5^+^ CMs (Protze et al., 2017), indicating preferential differentiation toward SAN head. The outcome of the two protocols might, however, be influenced not only by the different signaling modulators used but also by different culture conditions (two-dimensional [2D] versus three-dimensional [3D]).

All in all, a better understanding of the signaling pathways underlying SAN development including the association between BMP, RA, and WNT signaling would provide opportunities for improving differentiation efficiencies and obtain desired cellular fractions from hPSCs. Importantly, methods to differentiate cells to specific subpopulations of the SAN, such as the head, tail, and transition zone, will enable the creation of advanced cellular models relevant for disease modeling and drug screenings as well as informing the application of these cell types for therapeutic purposes.

## RA in epicardial development

The epicardium develops from a transient structure called the proepicardium, located at the venous pole of the developing heart. The proepicardium is characterized by the ubiquitous expression of *Tbx18*, *Tcf21*, and *Wt1* among other genes (Lupu et al., 2020). The precise origins of the proepicardium are poorly understood, and recent insights reveal that it receives contributions from multiple progenitor populations (Mommersteeg et al., 2010; Tyser et al., 2021; Zhang et al., 2021). It has been identified that a population anterior to the FHF has the potential to form myocardial components such as left ventricle, atrioventricular canal, and proepicardium (Tyser et al., 2021; Zhang et al., 2021). Similarly, tetramethylindocarbocyanine (DiI) labeling in chick and mouse embryos has shown that a *Tbx18*^+^ population lateral in the pSHF contributes to both the proepicardium and the sinus venosus myocardium (van Wijk et al., 2009; Mommersteeg et al., 2010). Although RA signaling in the pSHF is necessary for the differentiation of atrial and sinus venosus myocardium, its requirement for the formation of the proepicardium is not known. As depicted in Figure 2, Raldh2 expression begins in the proepicardium and is also found in the epicardium at E12.5. Loss of *Raldh2* does not impair the formation of proepicardium itself. However, ventricular growth and formation of coronary vasculature were severely affected in *Raldh2*-deficient mice (Niederreither et al., 2001; Lin et al., 2010).

RA signaling in the epicardium is regulated by WT1 through direct activation of *Raldh2* (Guadix et al., 2011). *Raldh2* is expressed in a spatiotemporal pattern similar to that of *Wt1*, and structural defects in mice lacking either gene were found to be similar (Perez-Pomares et al., 2002; Guadix et al., 2011; von Gise et al., 2011). *Raldh2* levels in *Wt1* knockout mice were more severely reduced in ventricular epicardium compared with atrial epicardium (Guadix et al., 2011). Besides *Wt1*, another important proepicardial and epicardial gene, *Tcf21*, is also associated with RA signaling. *Tcf21* is induced by RA treatment, which promotes fibroblast differentiation at the expense of smooth muscle cells (Acharya et al., 2012; Braitsch et al., 2012). Similarly, in Dhrs3^−/−^ hearts with increased RA synthesis, an increase in fibroblasts was observed (Xiao et al., 2018). *In vivo* findings thus suggest that RA may not be required for proepicardial specification itself but that it has a critical role in the subsequent development of the epicardium and further differentiation toward fibroblasts.

**Figure 5:**
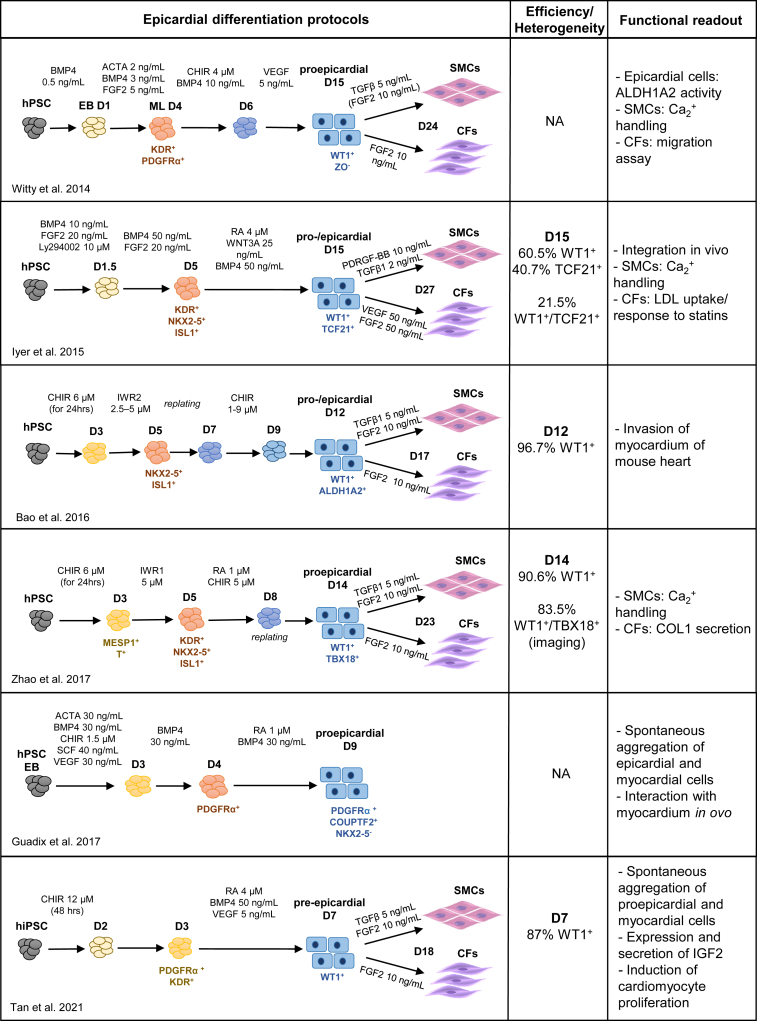
Summary of differentiation protocols for the generation of (pro)epicardial cells from hPSCs

**Figure 6:**
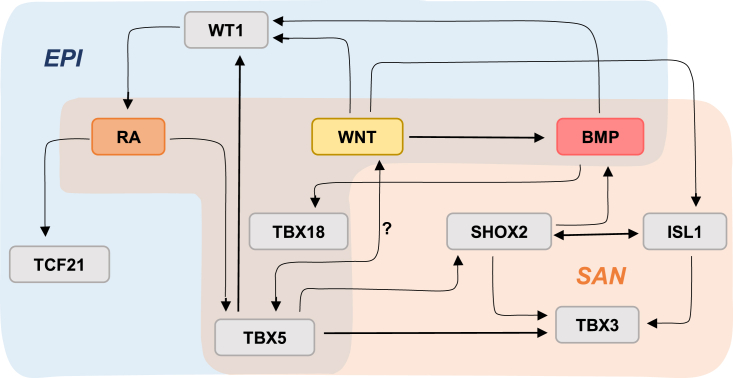
Transcriptional networks involved in epicardial (EPI) and sinoatrial node (SAN) development RA, WNT, and BMP signaling are implicated in EPI (blue) and SAN (orange) specification. Both TBX18 and TBX5 are expressed in EPI and SAN progenitors where they regulate a number of cell-type-specific target genes.

In contrast to *in vivo* findings, *in vitro* studies show that treatment with RA steers proepicardial differentiation of hPSCs (Iyer et al., 2015; Guadix et al., 2017; Zhao et al., 2017; Tan et al., 2021). Manipulation of BMP, WNT, RA signaling pathways, or a combination of these allowed the generation of epicardial-like cells from hPSCs (Figure 5) (Witty et al., 2014; Iyer et al., 2015; Bao et al., 2016; Guadix et al., 2017; Zhao et al., 2017). In a more recent protocol, BMP4 + RA + vascular endothelial growth factor (VEGF) was used to generate proepicardial cells; however, the added advantage of including VEGF as opposed to using BMP4 + RA alone was not investigated (Tan et al., 2021). Like RA, WNT signaling is also only implicated in later stages of epicardial development, epicardial EMT, and formation of coronary vessels *in vivo* (Zamora et al., 2007; von Gise et al., 2011). However, there is overwhelming evidence implicating *Bmp4* in proepicardial development as well as epicardial migration (Schlueter et al., 2006; Ishii et al., 2010; Liu and Stainier, 2010). As discussed above, RA, BMP, and WNT pathways are also implicated in SAN differentiation, reflecting shared ontogeny with proepicardial cells (van Wijk et al., 2009; Mommersteeg et al., 2010) (Figure 6). Interestingly, the common link between RA, WNT, and BMP pathways appears to be *TBX5*. Besides its role in the pSHF, *Tbx5* is also implicated in proepicardial development and epicardial migration (Liu and Stainier, 2010; Diman et al., 2014). Proepicardial cells generated from hPSCs *in vitro* using BMP4 + RA also express *Tbx5* (Tan et al., 2021). Moreover, microarray analysis of Tbx5-induced genes identified *Wt1* as a possible target (Plageman and Yutzey, 2006).

Timing of application and/or concentration of signaling modulators appears to delineate SAN versus epicardial differentiation. In about half of the epicardial differentiation protocols, signaling modulators are introduced at the cardiac progenitor stage marked by NKX2–5 and ISL1 expression (Iyer et al., 2015; Bao et al., 2016; Zhao et al., 2017). This is in accordance with *in vivo* findings that revealed the contribution of NKX2–5^+^/ISL1^+^ cardiac progenitors to the proepicardium (Zhou et al., 2008). In other protocols, an earlier population at the cardiac mesoderm stage marked by PDGFRα was steered toward the epicardial lineage (Witty et al., 2014; Guadix et al., 2017; Tan et al., 2021), implying that there are multiple differentiation routes toward epicardial cells. Whether these correspond to the different progenitor populations as shown *in vivo* is yet to be evaluated (Mommersteeg et al., 2010; Tyser et al., 2021; Zhang et al., 2021).

Activation either of canonical WNT signaling alone (Bao et al., 2016) or in combination with BMP4 (Witty et al., 2014) or RA (Zhao et al., 2017) or both BMP4 and RA (Iyer et al., 2015; Tan et al., 2021) was used in these methods (Figure 5). Inhibition of canonical WNT signaling resulted in a decrease in *WT1*, indicating that at least endogenous WNT signaling is required for epicardial differentiation (Witty et al., 2014; Iyer et al., 2015). The differences in the choice of signaling molecules and the timing of application likely explains the variations in efficiency and heterogeneity observed in the resulting epicardial populations (Figure 5). The percentage of WT1^+^ cells was greater (>90%) in protocols that used either WNT activation alone (Bao et al., 2016) or WNT + RA (Zhao et al., 2017). In the WNT + BMP4 + RA protocol (Iyer et al., 2015), only 20% of the end population was positive for both WT1 and TCF21. On the contrary, combined treatment of WNT + RA yielded 80% of cells positive for both WT1 and TBX18 (Zhao et al., 2017). Similarly, treatment with BMP4 + RA + VEGF yielded a high percentage of proepicardial cells that co-expressed WT1 and ZO1 (Tan et al., 2021). As shown in a recent study in the mouse heart, typical epicardial markers are co-expressed in the proepicardium and epicardium until E13.5, as opposed to the septum transversum, where a more heterogeneous expression of the same markers is found (Lupu et al., 2020). This finding suggests that better characterization is needed in order to determine the identity of WT1^+^ cells *in vitro*.

An important criterion for assessing the functionality of hPSC-derived epicardial cells is their ability to generate epicardial-derived cell populations (EPDCs). Epicardial-derived progenitor cells undergo EMT and give rise to EPDCs such as fibroblasts and smooth muscle cells (Quijada et al., 2020). These cell types are valuable for disease modeling and tissue engineering applications aimed at reconstructing the microenvironment of the heart. Epicardial cells generated from most protocols *in vitro* undergo EMT and further differentiate to smooth muscle cells and fibroblasts upon manipulation of transforming growth factor beta (TGFB) and fibroblast growth factor (FGF) pathways, respectively (Witty et al., 2014; Iyer et al., 2015; Zhao et al., 2017; Tan et al., 2021). Furthermore, epicardial cells and cardiac fibroblasts generated from such two-step protocols are being implemented in disease modeling, in maturation of hPSC CMs, and even in augmenting heart regeneration after myocardial infarction (Bargehr et al., 2019; Giacomelli et al., 2020; Tan et al., 2021). Collectively, data from the various differentiation approaches suggest that there are multiple strategies to obtain epicardial cells from hPSCs, which induce a transcriptional cascade involving *WT1*, *RALDH2*, and *TBX18*. However, additional insight into the heterogeneity in the resulting populations, as well as a uniform set of markers and parameters used for evaluation of epicardial and EPDC phenotypes, must still be established.

## RA in ventricular specification and maturation

The FHF and the aSHF give rise to the left and right ventricles, respectively (Zaffran et al., 2004; Verzi et al., 2005). The majority of ventricular cardiomyocytes are derived from Mesp1^+^ cardiac progenitors (Saga et al., 1999). More recently, it has been found that derivatives of two temporally distinct *Foxa2*^+^ progenitor populations also contribute to 30%–50% of ventricular cardiomyocytes (Bardot et al., 2017; Ivanovitch et al., 2021). Whether these Foxa2^+^ cells constitute a subfraction of Mesp1^+^ progenitors or an altogether independent population remains to be determined. In the absence of RA signaling in mice, development of FHF-derived left ventricle proceeds normally (Ryckebusch et al., 2008). In zebrafish, however, RA-deficient embryos present significant expansion of a differentiating FHF population accompanied by a reduction in aSHF progenitors (Duong et al., 2021). In the SHF, RA maintains the border between aSHF and pSHF progenitors. Right ventricular progenitors located in the aSHF are demarcated by the expression of *Tbx1*. Tbx1 positively regulates *Cyp26a1–c1* genes involved in RA degradation, thereby antagonizing RA (Roberts et al., 2006). Expectedly, the treatment of pregnant mice at E7.75–E8.25 with a pan-RAR antagonist did not affect the right ventricle of the examined embryos (De Bono et al., 2018). Moreover, exogenous RA resulted in a hypoplastic ventricle by impairing aSHF differentiation toward the ventricular lineage (Bardot et al., 2017; Gonzalez et al., 2021). Similarly, in zebrafish, RA-induced *nr2f* genes were responsible for restricting ventricular CM numbers while promoting pharyngeal muscle development (Duong et al., 2018). Altogether, these findings suggest that RA is not required and is even detrimental for ventricular specification. In contrast, strong evidence has underscored the requirement of RA in later stages of ventricular development and maturation.

At E9.5 of mouse development, the ventricular wall comprises a very thin layer of myocardium. Growth of the ventricles, which involves trabeculation and subsequent thickening of the compact layer, requires proliferative and other morphogenic signals that are initiated around E9.5–E10.5. A majority of the paracrine signals that drive ventricular compact wall expansion are thought to be derived from the epicardium (Sucov et al., 2009). Epicardial migration begins at E9.5 and entirely covers the outer surface of the heart by E10.5 (Viragh and Challice, 1981), coinciding with ventricular expansion. In *Raldh2* knockout mouse embryos, abnormal looping results in a single ventricle, which lacks trabeculae (Niederreither et al., 1999). Ventricular hypoplasia was also observed in vitamin A-deficient rat progeny (Wilson and Warkany, 1949; Niederreither et al., 2001). Subsequent studies showed that the effect of RA on the ventricles is mediated by RXRα receptors in the epicardium (Merki et al., 2005). Moreover, ventricular defects observed in germline *Rxrα* knockout and *Raldh2* knockout embryos resemble the ventricular phenotype caused by defective epicardium (Takahashi et al., 2014).

In the majority of hPSC differentiation studies, obtained cardiomyocytes are a heterogeneous mix of various cardiac subtypes, with ventricular cardiomyocytes being the predominant group (Blazeski et al., 2012; Cyganek et al., 2018; Friedman et al., 2018). It is worth noting that in the absence of RA or other posterior fate-inducing signals, the default differentiation path of *MESP1*^+^ cardiac mesoderm cells *in vitro* is toward the ventricular lineage in the majority of cell lines and existing differentiation protocols. The RA-degrading enzyme *CYP26A1* is expressed at day 3 of differentiation, coinciding with peak *MESP1* expression, and is more suited for differentiation toward ventricular cardiomyocytes (Lee et al., 2017; Calderon et al., 2021; H.D.D., unpublished observations). Furthermore, treatment with an RA antagonist during differentiation, presumably at the cardiac mesoderm stage (day 3) or cardiac progenitor stage (day 5), steered cells toward a ventricular fate (Zhang et al., 2011; Tan et al., 2021).

It is well known that PSC-derived cardiomyocytes exhibit immature structural, metabolic, and electrophysiological properties, corresponding to fetal rather than adult cardiomyocytes. The effect of RA on maturation of PSC-derived cardiomyocytes has not yet been assessed. Nevertheless, as the effect of RA is mediated by paracrine factors from the epicardium such as insulin-like growth factor (IGF) and FGF, approaches that recapitulate epicardial-myocardial interactions have been probed. Co-culture of hPSC-derived epicardial cells with cardiomyocytes in 2D cultures as well as 3D constructs enhanced contractility and calcium handling properties of the cardiomyocytes. It was shown that the proliferative effect of proepicardial cells on co-cultured ventricular cardiomyocytes was mediated at least in part by RA-dependent IGF2 signaling (Tan et al., 2021). IGF2 has also been utilized for the maturation of compact ventricular cells derived from hPSCs (Funakoshi et al., 2021). IGF2 was strongly decreased in both Raldh2^−/−^ and *Rxrα* mutants and is implicated in ventricular wall proliferation (Brade et al., 2011; Li et al., 2011). Furthermore, several studies have shown that IGF1 in combination with thyroid hormone T3 and dexamethasone or neuregulin promotes maturation of hPSC-derived cardiomyocytes in 2D and 3D cultures (Birket et al., 2015; Rupert and Coulombe, 2017; Huang et al., 2020). A head-to-head comparison of the direct effects of secreted epicardial factors versus co-culture with epicardial cells or epicardial-derived fibroblasts on hPSC-derived cardiomyocytes is warranted for identification of the best strategies to promote maturation *in vitro*.

## Concluding perspectives

**Figure 7:**
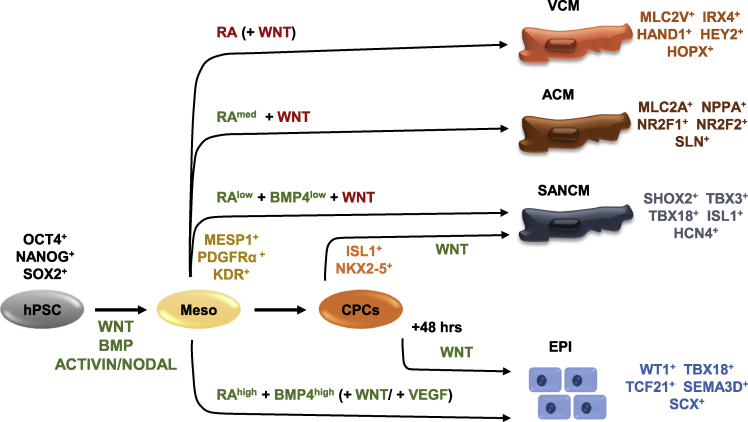
Schematic overview of the differentiation of various cardiac cell types from hPSCs Signaling pathways implicated in each step are indicated in green (activation) or red (inhibition). Genes that are typically used as markers for each cell type are also mentioned. The majority of the protocols rely on modulation of signaling pathways at the cardiac mesoderm stage, marked by MESP1, PDGFRα, or KDR. Inhibition of RA signaling in combination with inhibition of WNT or inhibition of WNT alone gives rise to VCM. Activation of RA signaling (using intermediate concentrations of 0.5–1 μM RA) in conjunction with inhibition of WNT signaling generates ACM. Activation of RA (using lower concentrations of 0.25 μM RA) along with activation of BMP signaling and inhibition of WNT signaling yields SANCM. SANCM can also be generated by activating WNT signaling at the cardiac progenitor stage, marked by ISL1 and NKX2–5. Replating ISL1^+^/NKX2–5^+^ cardiac progenitors and subsequent activation of WNT signaling results in (pro)epicardial cells. Alternatively, activation of RA signaling (using higher concentration of 1–4 μM RA) alongside activation of BMP and WNT/VEGF signaling also gives rise to (pro)epicardial cells. ACM, atrial-like cardiomyocytes; CPC, cardiovascular progenitor cells; EPI, proepicardial/epicardial cells; hPSCs, human pluripotent stem cells; KDR, kinase insert domain receptor; Meso, mesoderm; SANCM, sinoatrial nodal-like cardiomyocytes; VCM, ventricular-like cardiomyocytes.

Cardiac development is a complex process that relies on coordinated spatiotemporal interactions of signaling pathways, transcriptional regulators, and epigenetic modifiers. As presented in this review, RA signaling is indispensable in cardiogenesis for the development of atria and sinus venosus during early embryogenesis and for ventricular growth and expansion at later stages. *In vitro*, RA is utilized in the differentiation of multiple cardiac cell types from hPSCs (Figure 7). Addition of RA to atrial and sinoatrial differentiations from hPSCs mimics the earliest stages of heart development synonymous with the expression of *Raldh2* in pSHF progenitors. Timing and concentration of signaling modulators are crucial for successful differentiation of the desired cardiac cell type. Whereas SANCM differentiation uses lower concentrations of BMP4 (2.5 ng/mL) and RA (0.25 μM), epicardial cell specification is induced by higher concentrations of BMP4 (10–50 ng/mL) and RA (1–4 μM). It is worth noting that the important difference in the SAN and epicardial differentiation protocols, which use canonical WNT signaling alone, is the timing of application (Bao et al., 2016; Ren et al., 2019). In SAN differentiation, WNT signaling is activated at day 5 upon the expression of NKX2–5. In epicardial differentiation, on the other hand, cells are replated and cultured for 48 h prior to WNT activation. For the differentiation of atrial cells from hPSCs, activation of RA signaling is the only known strategy thus far. The majority of the studies demonstrate that application of RA (0.5–1 μM) shortly after mesoderm induction directs hPSCs toward an atrial fate.

Furthermore, aforementioned *in vitro* studies point to a concerted action of RA, BMP, and WNT pathways in the specification of SANCMs and epicardial cells. Interestingly, BMP and WNT signaling co-operatively induce *Hox* gene expression required for the specification of hematopoietic cells from ventral mesoderm (Lengerke et al., 2008). Similarly, WNT-dependent activation of *Hox* genes has been reported in the early embryo (Neijts et al., 2016). Whether a similar mechanism could explain the findings in the context of cardiac differentiations, in which BMP and/or WNT are used in lieu of RA, is yet to be determined. Moving forward, studies to better define the hierarchy and interaction between HOX, TALE family members such as MEIS1/2, NR2F transcription factors, and TBX5 will shed light on the molecular mechanisms underlying RA-driven cardiac cell specification. In particular, tissue-specific co-factors and targets of TBX5, which discern epicardial, atrial, or sinoatrial lineage identity, are to be investigated. Directed differentiation of hPSCs to cardiac cell types will serve as an excellent model in this context. Sampling of cells at various time points during differentiation and comprehensive analysis of the genetic and epigenetic changes will unravel cell-type-specific gene-regulatory networks. Such insights will in turn inform further improvement of *in vitro* differentiation protocols and even trigger the development of systems and synthetic biology approaches to precisely modulate cell fate in a dish.

## Author contributions

Conceptualization, data curation, and writing, A.W. and H.D.D.; visualization, A.W.; review and editing, H.D.D., V.M.C., and G.J.J.B.; supervision, H.D.D.

## Conflicts of interest

G.J.J.B. is a co-founder of Pacingcure.
